# Psychological Distress and Self-Management in CKD: A Cross-Sectional Study

**DOI:** 10.1016/j.xkme.2023.100712

**Published:** 2023-08-11

**Authors:** Cinderella K. Cardol, Yvette Meuleman, Henriët van Middendorp, Paul J.M. van der Boog, Luuk B. Hilbrands, Gerjan Navis, Yvo W.J. Sijpkens, Jacob K. Sont, Andrea W.M. Evers, Sandra van Dijk, E-GOAL Study Group

**Affiliations:** 1 Health, Medical and Neuropsychology Unit, Leiden University, Leiden, The Netherlands; 2 Department of Clinical Epidemiology, Leiden University Medical Center, Leiden, The Netherlands; 3 Department of Nephrology, Leiden University Medical Center, Leiden, The Netherlands; 4 Department of Nephrology, Radboud University Medical Center, Nijmegen, The Netherlands; 5 Department of Nephrology, University Medical Center Groningen, Groningen, The Netherlands; 6 Department of Internal Medicine, Haaglanden Medical Center Bronovo, The Hague, The Netherlands; 7 Department of Biomedical Data Sciences, Section Medical Decision Making, Leiden University Medical Center, Leiden, The Netherlands; 8 Medical Delta, Leiden University, TU Delft, and Erasmus University Rotterdam, The Netherlands

**Keywords:** Chronic kidney disease (CKD), kidney transplantation, psychological distress, self-management, lifestyle, adherence

## Abstract

**Rationale & Objective:**

Patients with chronic kidney disease (CKD) not receiving dialysis, including kidney transplant recipients, often experience difficulties regarding self-management. An important barrier for adherence to self-management recommendations may be the presence of psychological distress, consisting of depressive and anxiety symptoms. We investigated relationships between psychological distress and adherence to self-management recommendations.

**Study Design:**

Cross-sectional online questionnaire data as part of the E-GOAL study.

**Setting & Participants:**

Patients with CKD (estimated glomerular filtration rate, 20-89 mL/min/1.73 m^2^) were recruited from April 2018 to October 2020 at 4 hospitals in The Netherlands and completed online screening questionnaires.

**Exposures:**

Psychological distress, depressive symptoms, and anxiety symptoms.

**Outcomes:**

Dietary adherence, physical activity, medication adherence, smoking, body mass index, and a CKD self-management index (ie, the sum of 5 binary indicators of nonadherence to the recommended self-management factors).

**Analytical Approach:**

Adjusted multivariable regression and ordinal logistic regression analyses.

**Results:**

In our sample (N = 460), 27.2% of patients reported psychological distress, and 69.8% were nonadherent to 1 or more recommendations. Higher psychological distress was significantly associated with poorer dietary adherence (β^adj^, −0.13; 95% CI, −0.23 to −0.04), less physical activity (β^adj^, −0.13; 95% CI, −0.22 to −0.03), and lower medication adherence (β^adj^, −0.15; 95% CI, −0.24 to −0.05), but not with smoking and body mass index. Findings were similar for depressive symptoms, whereas anxiety was only associated with poorer dietary and medication adherence. Every 1-point higher psychological distress was also associated with a higher likelihood of being nonadherent to an accumulating number of different recommendations (adjusted OR, 1.04; 95% CI, 1.02-1.07).

**Limitations:**

Cross-sectional design, possible residual confounding, and self-report.

**Conclusions:**

Many people with CKD experience psychological distress, of whom most have difficulties self-managing their CKD. Given the relationship between psychological distress and adherence to CKD self-management recommendations, behavioral interventions are needed to identify and treat psychological distress as a potential barrier to CKD self-management.

**Plain-Language Summary:**

This online questionnaire study investigated relationships between psychological distress and self-management among 460 people with chronic kidney disease. Over a quarter of them reported mild-to-severe psychological distress. Alarmingly, 4 out of 5 patients with psychological distress were also nonadherent to 1 or more self-management recommendations, and higher levels of psychological distress were associated with poorer dietary and medication adherence and lower physical activity. Moreover, patients who suffered from moderate-to-severe distress were relatively more often nonadherent to 3 or more recommendations compared with patients with no or mild distress symptoms. So, it seems that psychological distress can be a barrier for self-management. To support patients in managing chronic kidney disease, researchers and health professionals should not overlook patients’ mental health.

From the diagnosis of chronic kidney disease (CKD) onwards, patients are confronted with profound changes that require extensive emotional skills, including coping with the diagnosis, affected future perspectives, physical symptoms, and social implications.^1^ An additional burden is adhering to disease self-management recommendations in order to decelerate disease progression and reduce risks of adverse health outcomes.^2^ CKD is both impactful and demanding, and requires significant emotional and behavioral management.

Behavioral CKD management comprises adherence to general and disease-specific dietary prescriptions,^3^^,^^4^ regular physical activity,^5^ medication-taking as prescribed,^6^^,^^7^ and no tobacco smoking.^8^^,^^9^ Weight management is also included in CKD guidelines by the proxy body mass index (BMI).^10^ Despite the beneficial health outcomes associated with most self-management behaviors, in recent studies among patients with CKD not receiving dialysis, ∼78% of patients had a suboptimal diet, 34%-47% reported limited physical activity, 12%-67% were nonadherent to medication prescriptions, and 13%-17% were current smokers.^9^^,^^11^^,^^12^

High nonadherence rates may be partly related to the emotional impact of the disease, as reflected by high prevalence of psychological distress, which affects 21%-34% of patients.^13^^,^^14^ Psychological distress is a negative emotional response to chronic disease stressors,^15^^,^^16^ commonly assessed as depressive and anxiety symptoms in a composite measure or separately.^16^, ^17^, ^18^ Distress is associated with adverse health outcomes, including accelerated CKD progression and mortality.^14^^,^^19^ One explanation for these adverse outcomes is that distress can be a barrier for adherence.^14^ Moreover, increasing psychological distress levels may hinder an increasing number of different nonadherent behaviors, which could lead to even worse health outcomes for patients who suffer from severe distress levels.^11^^,^^20^

The relationship of psychological distress with self-management has been examined in other chronically ill populations. For instance, patients with diabetes and distress were more likely to report dietary nonadherence, physical inactivity, and tobacco smoking than patients without distress.^21^^,^^22^ Although there are many studies of patients treated with dialysis,^23^ few studies address patients with CKD not receiving dialysis. Additionally, most published studies examined only 1 or a few self-management factors, involved relatively small samples, or focused only on symptoms of depression such as hopelessness. For example, higher levels of hopelessness were associated with several self-management measures, including a higher perceived burden of dietary restrictions, in a cross-sectional study among 461 patients, of whom over a quarter were not receiving dialysis.^24^ Recently, Choi et al^11^ found that a lack of physical activity and current smoking were significantly associated with higher psychological distress levels among patients with CKD. Their findings indicated that, compared with optimal self-management adherence, a higher number of nonadherent behaviors was linked to a greater risk of more severe distress. The authors emphasized that associations may also work the other way around, with distress hindering self-management.^11^ Therefore, the first aim of our study was to assess the relationship of psychological distress, and specifically depressive and anxiety symptoms, with adherence to CKD self-management recommendations, including dietary adherence, physical activity, medication adherence, weight maintenance, and non-smoking.^2^ The second aim was to examine whether higher psychological distress levels, and specifically depressive and anxiety symptoms, are proportionately related to nonadherence to a higher number of different self-management recommendations.

## Methods

### Study Design

Cross-sectional survey data were collected for the *E*-health *G*uidance in identifying and *O*vercoming psychological barriers for *A*dopting a healthy *L*ifestyle among patients with CKD study (E-GOAL; Netherlands Trial Registry: NL7338), a multicenter randomized controlled trial to evaluate an electronic health care pathway. For the current study, we used baseline data from screening questionnaires completed by patients to examine eligibility for trial participation. The study was approved by the Medical Ethics Committee of Leiden University Medical Center (P17.172) and complies with the 1964 Declaration of Helsinki. The Strengthening the Reporting of Observational Studies in Epidemiology statement was used as a reporting reference.^25^

### Setting and Participants

**Figure 1:**
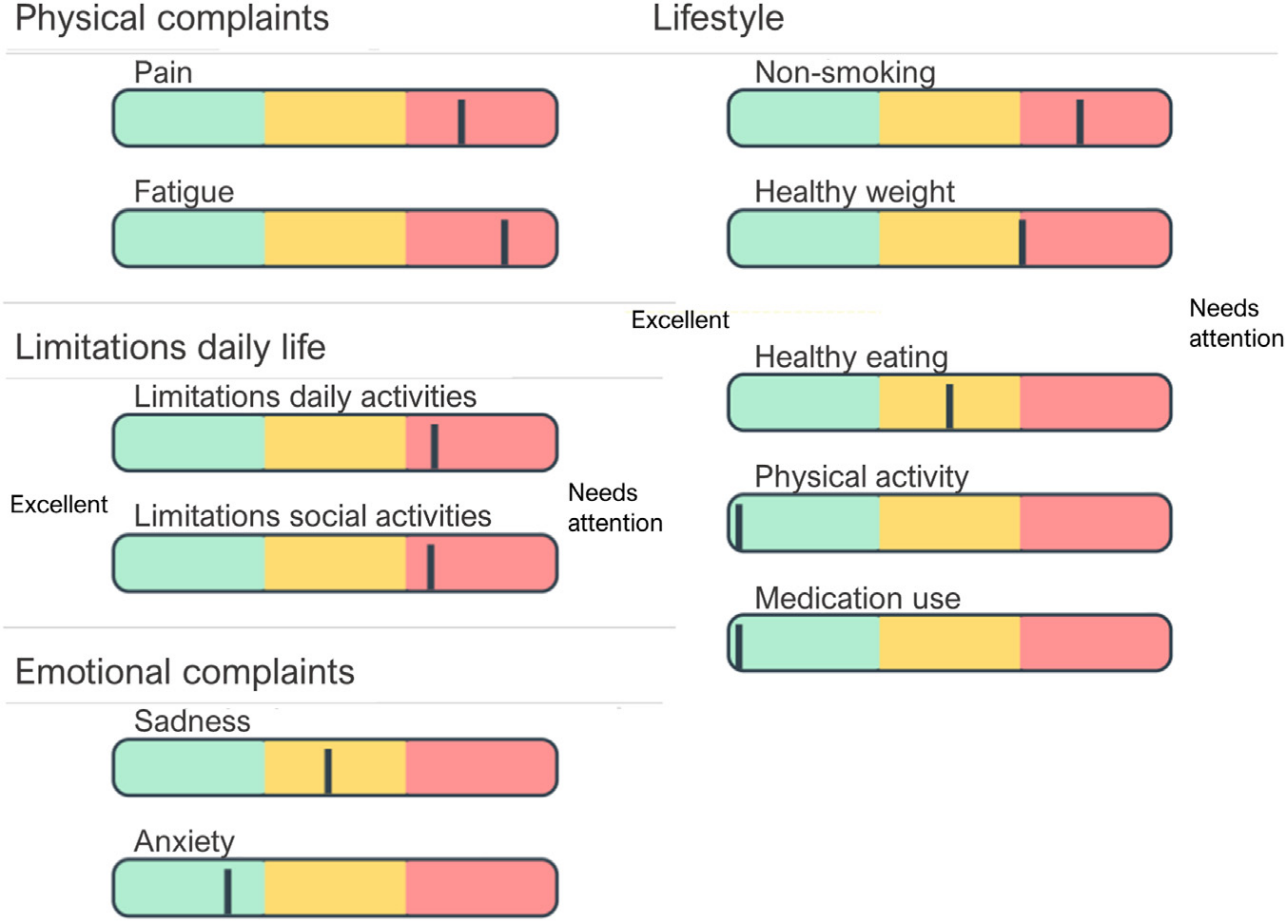
Example of personal profile charts. Traffic light colors indicate current status on domains of functioning and self-management. Additional explanations are shown when hovering the mouse cursor over a domain. This patient with chronic kidney disease shows moderate depressive symptoms (which may be influenced by severe physical complaints and limitations in daily life), heavy smoking, obesity, and moderate dietary adherence.

From April 2018 to October 2020, data were collected in 4 Dutch hospitals: Leiden University Medical Center, University Medical Center Groningen, Radboud university medical center, and Haaglanden Medical Center. Eligible patients were ≥18 years old, had an estimated glomerular filtration rate of 20-89 mL/min/1.73 m^2^, were treated by a nephrologist, and spoke Dutch. Exclusion criteria were rapid progression of kidney function loss (>10% over the last year), dialysis treatment or an expected need for kidney replacement therapy during the study (<6 months), kidney transplantation <1 year ago, systolic blood pressure <95 mmHg not reacting to antihypertensive medication cessation, or other problems likely to interfere with study participation (eg, progressive cancer, recent cardiovascular event, severe psychiatric disorders, problems in understanding written communication, or pregnancy). Patients received invitations to participate via their nephrologist during hospital visits or by mail, containing written information regarding study procedures and an informed consent form. Upon completing the written informed consent, patients received a link by email to online screening questionnaires in the application PatientCoach (www.patientcoach.lumc.nl). Paper-and-pencil questionnaires were available for individuals who had difficulties with online questionnaire completion. After completion (estimated duration, 5-15 min), participants could review personal profile charts (Fig 1), which displayed their results in the application, and receive paper versions by mail. Recruitment took place until the trial sample size was reached.^26^

### Measurements

#### Demographic and Clinical Characteristics

Questionnaires included demographic (age, sex, birth country, marital status, parenthood, education level, and employment status), disease, and treatment characteristics (comorbidities, treatment history for psychological and physical complaints), and health-related quality of life (HRQoL) measured with the RAND 36-item Short Form health survey.^27^ Physical and mental HRQoL component summary scores are shown as *T-*scores (Hays norm-based scoring algorithm; mean, 50 ± 10 [SD] in the general population), with higher scores indicating better HRQoL.^27^ The number of comorbidities was based on self-reported presence of hypertension, diabetes mellitus, cardiovascular disease, cancer, pulmonary, rheumatic, liver, gastrointestinal, or blood disease, and chronic fatigue syndrome. Additional medical data were extracted from hospital information systems (history of kidney transplantation or dialysis, estimated glomerular filtration rate (CKD-EPI [Chronic Kidney Disease Epidemiology Collaboration] creatinine equation), and office systolic and diastolic blood pressure).

#### Psychological Distress

Psychological distress was measured with the Patient Health Questionnaire Anxiety and Depression Scale (PHQ-ADS),^17^ a 16-item composite measure of depressive and anxiety symptoms. The scale consists of the 9-item Patient Health Questionnaire depression scale (PHQ-9)^28^ and the 7-item Generalized Anxiety Disorder scale (GAD-7),^29^ which are well-validated and commonly used among chronic disease populations, including CKD.^19^^,^^30^ Participants were asked to what degree a range of symptoms had bothered them over the past 2 weeks, with response options from 0 (not at all) to 3 (nearly every day). Total PHQ-ADS scores comprise the sum of PHQ-9 (range, 0-27) and GAD-7 (range, 0-21) ranging from 0-48, with higher scores indicating higher psychological distress levels. Cut-off points 10, 20, and 30 indicate mild, moderate, and severe levels, respectively (cut-off points 5, 10, and 15 for separate PHQ-9 and GAD-7). Scores below 10, indicating no or minimal distress symptoms, are referred to as no presence of psychological distress. The PHQ-ADS was reliable in this study with Cronbach’s α = 0.91.

#### CKD Self-Management

Dietary adherence, keeping a healthy diet according to CKD guidelines or individual prescriptions, was assessed using 2 self-developed questions: “In the past week, how often have you kept a healthy diet? Think of a specific dietary regimen as prescribed by your health care provider. If you have not received dietary prescriptions, think of a healthy diet for people with CKD in general, such as restricted salt consumption.” With scores on a 5-point scale ranging from never to always, and “In the past week, how well do you believe you have kept a healthy diet?” on a 1-10 rating scale ranging from very badly to very well. A nonlinear principle components analysis was conducted to combine the 2 ordinal items and obtain a single summary variable (z-score) for perceived dietary adherence.^31^ The 1-dimension-solution had an eigenvalue >1, explained 85.75% of variance, with Cronbach’s α = 0.83.

To measure physical activity, the short questionnaire to assess health-enhancing physical activity^32^ was used. Respondents indicated days per week, minutes per day, and intensity of commuting activities, leisure time, household activities, and work or school physical activities. Total scores were calculated for weekly medium-to-high intensity physical activity in minutes.

Medication adherence was measured with the simplified medication adherence questionnaire^33^ comprising 4 dichotomous yes or no items and 2 items to quantify omissions. For the latter, >2 doses missed over the past week and >2 days of nonadherence during the past 3 months are considered nonadherent.^33^ In the original scale, participants were considered nonadherent when ≥1 item indicated nonadherence. To facilitate interpretation, we used pooled sum scores (range, 0-6), with higher scores indicating better adherence. Weight maintenance was assessed by BMI (self-reported weight and height).^34^ For smoking behavior, patients indicated whether they currently smoked (yes or no).

Finally, we generated a CKD self-management index (CSI) by summing 5 binary indicators of nonadherence to the recommended self-management factors: (1) dietary adherence z-score >1 SD below sample mean (ie, perceived adherence to a healthy diet in the past week never, seldom or half the time, rated ≤6 on the 1-10 scale, as indicated on the separate dietary adherence items); (2) physical activity <150 minutes per week; (3) medication adherence score 0-4; (4) current smoker; and (5) BMI <18.5 or ≥25 kg/m^2^. One point is assigned for each nonadherent behavior (range, 0-5). The CSI is similar to healthy lifestyle indices constructed in previous studies.^8^^,^^35^

### Statistical Analyses

Descriptive statistics for sample characteristics, scores of psychological distress, and nonadherence were summarized as mean ± SD for normally distributed continuous variables, median (interquartile range) for skewed continuous variables, and frequency (proportion) for categorical variables. Differences in demographic and clinical characteristics were examined between participants without and with mild-to-severe psychological distress, between completely adherent patients and patients who were nonadherent to ≥1 self-management recommendations, and between complete and incomplete cases, using independent samples *t* tests for continuous variables and χ^2^ tests for categorical variables. Incomplete cases (7.4%) were more often filled out on paper-and-pencil questionnaires than complete cases. To avoid loss of power and biased results, missing data were imputed using multiple imputation (10 repetitions) under the missing at random assumption.^36^

To examine the hypotheses that psychological distress would be associated with less dietary adherence, physical activity, and medication adherence, univariate (crude) and multivariable (adjusted) regression analyses were conducted with the self-management factors as dependent variables. The association with weight maintenance was examined without predefined hypotheses, since BMI is not a behavior itself.^10^ Assumptions were checked and logarithmic transformations applied for variables showing heteroscedasticity or strongly skewed errors, including psychological distress, depressive and anxiety symptoms, physical activity, medication adherence, and BMI. For the hypothesized association of psychological distress with the dichotomous dependent variable, smoking, binomial logistic regression analyses were conducted. All multivariable models were adjusted for potential confounders: age, sex (male or female), marital status (single or partner), education level (lower or higher), estimated glomerular filtration rate, and physical comorbidities (0, 1, 2, and ≥3). To test the hypothesis that higher distress severity would be proportionately associated with a higher number of different nonadherent behaviors, we performed ordinal logistic regression analysis with psychological distress as the independent and the ordinal CSI as the dependent variable. The CSI’s highest categories were merged into nonadherent to 3-5 recommendations because few participants scored in those categories. All analyses were repeated for depressive and anxiety symptoms separately. Assumptions for the binomial logistic regression and ordinal logistic regression analyses were met.

As sensitivity analysis, analyses were repeated without imputing missing data. Also, subgroup analyses of participants with and without a history of kidney transplantation were conducted. Statistical analyses were performed using SPSS 25.0 (IBM), and *P* values <0.05 were considered statistically significant.

## Results

### Sample Characteristics

Out of 2240 eligible patients, 460 completed screening questionnaires (a flow diagram is reported in the trial paper).^25^ Table 1 shows participant characteristics. The majority were male (62.4%) and 68.9% had received a kidney transplant. Ages ranged from 19.0-88.0 years. The mean estimated glomerular filtration rate was 50.4 ± 17.6 mL/min/1.73 m^2^.

**Table 1:** Patient Characteristics of Total Chronic Kidney Disease Sample and by Subgroups Without and With Psychological Distress

Characteristic	Total Sample (N = 460)	No Psychological Distress (n = 335)	≥Mild Psychological Distress (n = 125)
Socio-demographic characteristics			
Age, y	58.5 ± 12.5	59.7 ± 12.3	55.4 ± 12.6
Male sex, n (%)	287 (62.4)	217 (64.8)	70 (56.0)
Born in The Netherlands, n (%)	434 (94.3)	322 (96.1)	112 (89.6)
Married/partnered, n (%)	366 (79.6)	274 (81.8)	92 (73.6)
Having children, n (%)	330 (71.7)	243 (72.5)	87 (69.6)
Lower education,^a^^,^^b^ n (%)	228 (49.6)	162 (48.4)	66 (52.8)
Unemployed, n (%)	228 (49.6)	166 (49.6)	66 (52.8)
Disease and treatment characteristics			
Kidney transplant recipient, n (%)	317 (68.9)	230 (68.7)	87 (69.6)
Time since last kidney transplantation,^c^^,^^d^ y	9.6 ± 8.2	10.0 ± 8.7	8.4 ± 6.3
History of dialysis, n (%)	175 (38.0)	127 (37.9)	48 (38.4)
Multimorbidity^d^	222 (48.4)	153 (45.9)	69 (55.2)
Diabetes mellitus,^d^ n (%)	70 (15.2)	46 (13.7)	24 (19.2)
Cardiovascular disease,^d^ n (%)	70 (15.2)	46 (13.7)	24 (19.2)
Hypertension,^e^ n (%)	145 (31.5)	98 (29.3)	47 (37.6)
eGFR, mL/min/1.73 m^2^	50.4 ± 17.6	50.8 ± 17.6	49.2 ± 17.6
Office SBP, mm Hg^f^	133.6 ± 15.8	132.1 ± 15.5	137.5 ± 15.8
Office DBP, mm Hg^f^	77.9 ± 9.7	77.0 ± 9.6	80.1 ± 9.5
Treatment history for psychological complaints,^d^ n (%)	125 (27.2)	65 (19.4)	60 (48.0)
Current treatment for psychological complaints,^d^ n (%)	22 (4.8)	6 (1.8)	16 (12.8)
Physical HRQoL^e^	43.2 ± 10.8	46.4 ± 9.6	34.6 ± 9.0
Mental HRQoL^b^	48.3 ± 10.8	53.1 ± 7.3	35.8 ± 8.3

*Note:* Continuous variables are presented as mean ± SD for normally distributed variables and as median (interquartile range) for skewed variables.

Abbreviations: DBP, diastolic blood pressure; eGFR, estimated glomerular filtration rate; HRQoL, health-related quality of life; SBP, systolic blood pressure.

a Lower education includes primary, prevocational, and vocational education; higher education includes advanced secondary and tertiary education.

b Three unknown.

c Only for kidney transplant recipients, n = 316.

d One unknown.

e Two unknown.

f Twenty-six unknown.

Table 2 shows prevalence rates and mean or median scores on variables of interest for our study aims. The prevalence of mild-to-severe psychological distress was 27.2% (score range, 0-42); depressive symptoms were reported by 36.7% (score range, 0-25); anxiety symptoms by 23.3% (score range, 0-19); and 18.7% reported both depressive and anxiety symptoms. Compared with participants without psychological distress, patients with distress were more often born outside The Netherlands, were younger, reported more comorbidities, lower physical and mental HRQoL, higher blood pressure, and more often had a treatment history for psychological symptoms (Table 1).

**Table 2:** Prevalence and Scores of Psychological Distress and Chronic Kidney Disease Self-Management Variables

Variable	Values (N = 460)
Psychological distress	
Psychological distress symptoms	
Prevalence,^a^ n (%)	125 (27.2)
Mean, 0-48 score	7.0 ± 7.2
Depressive symptoms	
Prevalence,^a^ n (%)	169 (36.7)
Mean, 0-27 score	4.2 ± 4.1
Anxiety symptoms	
Prevalence,^a^ n (%)	107 (23.3)
Mean, 0-21 score	2.8 ± 3.6
Self-management	
Dietary adherence	
Prevalence nonadherence (z-score >1 SD below sample mean)	80 (17.4)
Mean, 1-10 score^b^	7.4 ± 1.9
Physical activity	
Prevalence nonadherence (<150 min/wk), n (%)^c^	37 (8.0)
Mean, h/wk^b^	18.1 ± 15.7
Medication adherence	
Prevalence nonadherence (score 0-4), n (%)	61 (13.3)
Mean, 0-6 score	5.5 ± 0.9
Body mass index	
Prevalence nonadherence (<18.5 or ≥25), n (%)	249 (54.1)
Mean, kg/m^2^	26.1 ± 4.6
Smoking	
Prevalence nonadherence (current smoker), n (%)	36 (7.8)
Median, units/d	0 (0)
CKD self-management index^c^^,^^d^	
0 (completely adherent)	137 (29.8)
1 (1∗nonadherent)	208 (45.2)
2 (2∗nonadherent)	88 (19.1)
3 (≥3∗nonadherent)	25 (5.4)

*Note:* Continuous variables are presented as mean ± SD for normally distributed variables and as median (interquartile range) for skewed variables.

Abbreviation: CKD, chronic kidney disease.

a Prevalence of mild to severe symptoms, that is, psychological distress scores ≥10, depressive, and anxiety scores ≥5.

b One unknown.

c Two unknown.

d Calculated by summing 5 binary indicators of nonadherence to the recommended self-management factors.

Regarding nonadherence, 17.4% of our patients reported having a suboptimal diet, 8.0% reported engaging in less physical activity than recommended, 13.3% were not fully adherent to medication prescriptions, 54.1% did not have a healthy BMI (1.3% underweight, 36.3% overweight, and 16.5% obese), and 7.8% were current smokers. In total, 321 patients (69.7%) were nonadherent to ≥1 self-management recommendations, of whom 208 were nonadherent to 1 recommendation, 88 to 2 recommendations, 21 to 3, 4 to 4, and none of the participants were nonadherent to all 5 recommendations. Participants who were nonadherent to ≥1 self-management recommendations had lower education levels, more comorbidities, lower physical and mental HRQoL, higher blood pressure, and had received psychological treatment more often than participants who were completely adherent (Table S1).

### Psychological Distress and CKD Self-Management

In Table 3, crude and adjusted linear regression analyses of psychological distress and self-management are shown. Higher psychological distress was significantly associated with poorer dietary adherence (β^adj^, −0.13; 95% Confidence Interval [CI], −0.23 to −0.04), less physical activity (β^adj^, −0.13; 95% CI, −0.22 to −0.03), and lower medication adherence (β^adj^, −0.15; 95% CI, −0.24 to −0.05). Psychological distress was not significantly related to higher BMI (β^adj^, −0.09; 95% CI, −0.01 to 0.18) or the likelihood that patients were current smokers (adjusted odds ratio [aOR], 1.03; 95% CI, 0.99-1.07).

**Table 3:** Linear Regression of Psychological Distress and Chronic Kidney Disease Self-Management

	Crude		Adjusted^a^	
	Coeff.^b^ (95% CI)	*P*	Coeff.^b^ (95% CI)	*P*
Dietary adherence	−0.19 (−0.28 to −0.10)	<0.001	−0.13 (−0.23 to −0.04)	0.006
Physical activity	−0.11 (−0.20 to −0.02)	0.02	−0.13 (−0.22 to −0.03)	0.01
Medication adherence	−0.19 (−0.28 to −0.10)	<0.001	−0.15 (−0.24 to −0.05)	0.002
Body mass index	0.11 (0.02 to 0.20)	0.02	0.09 (−0.01 to 0.18)	0.07
Smoking	1.03 (0.99 to 1.07)	0.20	1.04 (0.99 to 1.08)	0.13

Abbreviations: CI, confidence interval; Coeff., regression coefficient.

a Adjusted for age, sex, education level, marital status, comorbidities, and kidney function (estimated glomerular filtration rate).

b Beta for continuous dependent variables, odds ratio for dichotomous variable.

### Depressive and Anxiety Symptoms and CKD Self-Management

As shown in Tables 4 and 5, higher depressive and anxiety symptom levels were significantly associated with poorer dietary adherence (β^adj^, −0.14; 95% CI, −0.23 to −0.04 and β^adj^, −0.11; 95% CI, −0.20 to −0.01, respectively) and medication adherence (β^adj^, −0.15; 95% CI, −0.24 to −0.05 and β^adj^, −0.13; 95% CI, −0.22 to −0.03, respectively). Only reporting more depressive symptoms was associated with lower physical activity (β^adj^, −0.15; 95% CI, −0.25 to −0.06), whereas no significant association between anxiety and physical activity was observed (β^adj^, −0.07; 95% CI, −0.16 to 0.03). No significant associations were found for either depressive or anxiety symptoms with BMI (β^adj^, 0.08; 95% CI, −0.01 to 0.18 and β^adj^, 0.05; 95% CI, −0.05 to 0.14, respectively) or smoking (aOR, 1.07; 95% CI, 1.00-1.16 and aOR, 1.04; 95% CI, 0.95-1.14, respectively).

**Table 4:** Linear Regression of Depressive Symptoms and Chronic Kidney Disease Self-Management

	Crude		Adjusted^a^	
	Coeff.^b^ (95% CI)	*P*	Coeff.^b^ (95% CI)	*P*
Dietary adherence	−0.19 (−0.28 to −0.10)	<0.001	−0.14 (−0.23 to −0.04)	0.004
Physical activity	−0.14 (−0.23 to −0.05)	0.003	−0.15 (−0.25 to −0.06)	0.002
Medication adherence	−0.18 (−0.27 to −0.09)	<0.001	−0.15 (−0.24 to −0.05)	0.002
Body mass index	0.12 (0.03 to 0.21)	0.01	0.08 (−0.01 to 0.18)	0.09
Smoking	1.06 (0.99 to 1.14)	0.09	1.07 (1.00 to 1.16)	0.07

Abbreviations: CI, confidence interval; Coeff., regression coefficient.

a Adjusted for age, sex, education level, marital status, comorbidities, and kidney function (estimated glomerular filtration rate).

b Beta for continuous dependent variables, odds ratio for dichotomous variable.

**Table 5:** Linear Regression of Anxiety Symptoms and Chronic Kidney Disease Self-Management

	Crude		Adjusted^a^	
	Coeff.^b^ (95% CI)	*P*	Coeff.^b^ (95% CI)	*P*
Dietary adherence	−0.16 (−0.25 to −0.07)	0.001	−0.11 (−0.20 to −0.01)	0.03
Physical activity	−0.05 (−0.14 to 0.04)	0.28	−0.07 (−0.16 to 0.03)	0.19
Medication adherence	−0.17 (−0.26 to −0.08)	<0.001	−0.13 (−0.22 to −0.03)	0.009
Body mass index	0.06 (−0.03 to 0.15)	0.22	0.05 (−0.05 to 0.14)	0.35
Smoking	1.03 (0.94 to 1.12)	0.53	1.04 (0.95 to 1.14)	0.38

Abbreviations: CI, confidence interval; Coeff., regression coefficient.

a Adjusted for age, sex, education level, marital status, comorbidities, and kidney function (estimated glomerular filtration rate).

b Beta for continuous dependent variables, odds ratio for dichotomous variable.

### CSI

Table 6 presents distributions of adherence to self-management recommendations per psychological distress level. Taken together, 102 participants reported both mild-to-severe psychological distress and nonadherence to ≥1 self-management recommendations. Out of all participants who suffered from distress, 81.6% also had problems with self-management; out of all participants with self-management problems, 31.8% also had heightened psychological distress. Complete adherence to all 5 self-management recommendations was most common among patients without distress (34.0%) and least common among those with moderate-to-severe distress (11.8%). Inversely, nonadherence to ≥3 recommendations was least common in the no-distress group (3.9%) and most common in the moderate-to-severe distress group (14.7%). The distribution patterns of adherence per depressive or anxiety level were similar.

**Table 6:** Adherence to Chronic Kidney Disease Self-Management Recommendations by Psychological Distress, Depressive, and Anxiety Symptoms

Psychological Distress			
CSI,^a^ n (%)	No distress (n = 335)	Mild distress (n = 91)	Moderate-to-severe distress (n = 34)
0 (completely adherent)	114 (34.0)	19 (20.9)	4 (11.8)
1 (1∗nonadherent)	150 (44.8)	41 (45.1)	17 (50.0)
2 (2∗nonadherent)	56 (16.7)	24 (26.4)	8 (23.5)
3 (≥3∗nonadherent)	13 (3.9)	7 (7.7)	5 (14.7)
**Depressive symptoms**			
CSI,^a^ n (%)	No depression (n = 291)	Mild depression (n = 120)	Moderate-to-severe depression (n = 49)
0 (completely adherent)	100 (34.4)	32 (26.7)	5 (10.2)
1 (1∗nonadherent)	128 (44.0)	56 (46.7)	24 (49.0)
2 (2∗nonadherent)	51 (17.5)	24 (20.0)	13 (26.5)
3 (≥3∗nonadherent)	11 (3.8)	7 (5.8)	7 (14.3)
**Anxiety symptoms**			
CSI,^a^ n (%)	No anxiety (n = 353)	Mild anxiety (n = 81)	Moderate-to-severe anxiety (n = 26)
0 (completely adherent)	115 (32.6)	18 (22.2)	4 (15.4)
1 (1∗nonadherent)	156 (44.2)	39 (48.1)	13 (50.0)
2 (2∗nonadherent)	64 (18.1)	17 (21.0)	7 (26.9)
3 (≥3∗nonadherent)	16 (4.5)	7 (8.6)	2 (7.7)

Abbreviation: CSI, chronic kidney disease self-management index.

a Two unknown.

These observed accumulation patterns were confirmed by significant ordinal logistic regression analyses of the CSI by psychological distress: every 1-point higher psychological distress was associated with a 1.04 times higher likelihood of being nonadherent to an accumulating number of self-management recommendations (crude odds ratio, 1.05; 95% CI, 1.03-1.08 and aOR, 1.04; 95% CI, 1.02-1.07). Similar results were found for depressive (crude odds ratio, 1.11; 95% CI, 1.06-1.15 and aOR, 1.09; 95% CI, 1.04-1.14) and anxiety symptoms (crude odds ratio, 1.08; 95% CI, 1.03-1.13 and aOR, 1.06; 95% CI, 1.01-1.11) separately.

### Sensitivity Analyses

Tables S2-S5 show sensitivity analyses repeated on the original dataset. All results remained stable as compared with analyses conducted in the multiple imputation dataset. Results of subgroup analyses were similar for patients with and without a history of kidney transplantation, except for smoking: For patients who did not receive a kidney transplant (n = 143; 9.1% smokers), psychological distress was associated with a higher likelihood that patients were current smokers (aOR, 1.10; 95% CI, 1.02-1.18), whereas among kidney transplant recipients (n = 317; 7.3% smokers) there was no significant association (aOR, 1.00; 95% CI, 0.94-1.07).

## Discussion

This study shows that over a quarter of patients with CKD not receiving dialysis report psychological distress. Psychological symptoms are associated with poor health outcomes, including disease progression, diminished HRQoL, and even an increased mortality risk.^14^^,^^19^^,^^37^ Our findings support a potential explanation of the relationship between psychological distress and poor outcomes. Higher psychological distress, and its underlying depressive and anxiety symptoms, are associated with poorer adherence to several health-enhancing self-management recommendations. Moreover, patients with higher distress levels have a higher likelihood to be unsuccessful in multiple self-management areas compared with patients with lower distress. Psychological distress could thus hinder adequate CKD management.

Linear associations of psychological distress with a lack of physical activity and medication nonadherence observed in our study are similar to those described in literature.^11^^,^^38^ Additionally, our results show that higher distress is related to poorer dietary adherence. In contrast to recent research among patients with CKD,^11^ we did not find a clear association between psychological distress and smoking. This recent study had more participants, of whom 17.3% were current smokers,^11^ compared with 7.8% in our sample. We did observe a significant association between distress and smoking in a subgroup of patients without a history of kidney transplantation but not among kidney transplant recipients. However, since subgroups were somewhat small with very few smokers, these findings should be interpreted with caution.

Findings of depressive and anxiety symptoms separately are similar to psychological distress as a composite: Associations of distress with dietary and medication adherence can be explained by both depressive and anxiety symptoms, whereas having only more depressive symptoms is related to less physical activity. The main effects are slightly larger for depressive symptoms compared with anxiety symptoms. These findings suggest that depressive symptoms have a more important role in self-management than anxiety. A possible explanation may be that typical depressive symptoms are pessimistic perceptions and underestimations of one’s capabilities to engage in self-management, which could have discouraging effects. Alternatively, anxiety may work in 2 ways because of different coping styles, that is, avoidant or approach coping^39^: anxiety could have a paralyzing effect on health behavior but may also activate and motivate patients to be adherent.^40^ Although we did not find indications that higher anxiety would be associated with better adherence either, such contrasting responses could have been present within the sample and may have somewhat diluted associations.

Another explanation for the relatively small linear associations in general may be that self-management is not severely hampered if patients only experience mild psychological distress levels. Our findings suggest that the number of suboptimal self-management behaviors accumulates proportionally with distress severity. Patients who suffer from moderate-to-severe distress are relatively more often nonadherent to 3 or more recommendations compared with patients with no or mild distress. This is alarming because detrimental effects of nonadherence to multiple self-management recommendations may be additive.^8^^,^^41^

This study has some limitations. First, data are cross-sectional, making it impossible to determine relationships’ directionality. Plausibly, associations between psychological distress and self-management are bi-directional.^15^^,^^20^^,^^35^ On the one hand, psychological distress may entail negative cognitions and expectations, excessive focus on somatic symptoms, problems in motivation, energy, self-efficacy, concentration, or social withdrawal, which may all hinder patients’ ability to engage in healthy self-management; on the other hand, unhealthy behaviors may hamper psychological wellbeing through various mechanisms, for example, diminished social or physical activity, decreased fitness, or a lack of self-esteem owing to an inability to succeed in adherence.^15^^,^^20^^,^^42^ Future research with longitudinal designs should investigate the exact working mechanisms. Second, we adjusted for multiple potential confounders, including kidney function and multimorbidity. However, possible residual confounding from unmeasured variables should be considered.^43^ For example, we adjusted for education, but did not include other indicators of socioeconomic position, such as income.^44^ Third, self-management behaviors were measured by self-report, which could have overestimated or underestimated true adherence.^45^^,^^46^ The strengths of this study are the large sample size and relatively low percentage of missing data.^36^ Also, the high degree of multimorbidity of diabetes and cardiovascular diseases in our sample promotes generalizability to other patient populations.

The high prevalence of psychological and self-management difficulties and their interrelatedness emphasize a need for detection and treatment of both psychological distress and nonadherence to self-management recommendations in clinical practice. First, psychological distress and its constituents depression and anxiety are burdensome and important priorities for patients with CKD,^1^^,^^47^ but often remain unnoticed because patients and health care professionals may be hesitant to talk about psychological complaints and no regular assessments take place.^1^^,^^48^ Recognition is especially important since psychological distress could come along with self-management problems, as we observed that over 4 out of 5 patients with distress also reported nonadherence. Routine screening procedures comprising short questionnaires, such as the CSI developed in this study, may identify patients at risk of distress and inadequate self-management.^49^^,^^50^

Second, as psychological distress and self-management are associated, treating one could potentially improve the other. Intervention studies in other populations have shown that interventions focused on physical exercise and dietary improvements also reduced distress,^50^^,^^51^ and psychological interventions enhanced treatment adherence and reduced smoking.^52^ In terms of self-management models, for example, stress-coping models:^39^^,^^53^ when patients face disease-related stressors, strategies enhancing both problem-focused coping (facilitating self-management behaviors) and emotion-focused coping (regulating distress) should lead to the most beneficial outcomes.^16^ This implies that the effectiveness of self-management interventions for patients with CKD could be augmented by integrating cognitive-behavioral treatment of psychological distress.

To conclude, in the current study, we found that higher psychological distress is associated with poorer dietary and medication adherence and lower physical activity among patients with CKD not receiving dialysis. Furthermore, higher distress severity is associated with higher nonadherence risks to an increasing number of different self-management recommendations. These results suggest that psychological distress is a potential barrier for self-management. Tailored interventions to screen for and treat both psychological and self-management difficulties in parallel may be effective in improving physical and psychological outcomes.^20^^,^^22^^,^^35^ Future research should provide more insights into causality mechanisms in relationships of psychological distress, depressive symptoms, and anxiety symptoms with separate and concurrent self-management behaviors.

## Supplementary Material

Supplementary File (PDF) Item S1; Tables S1-S5.

## References

[1] de Jong Y., van der Willik E.M., Milders J. (2021). Person centred care provision and care planning in chronic kidney disease: which outcomes matter? A systematic review and thematic synthesis of qualitative studies: care planning in CKD: which outcomes matter?. BMC Nephrol.

[2] KDIGO CKD Work Group (2013). KDIGO 2012 clinical practice guideline for the evaluation and management of chronic kidney disease. Kidney Int Suppl.

[3] Garofalo C., Borrelli S., Provenzano M. (2018). Dietary salt restriction in chronic kidney disease: a meta-analysis of randomized clinical trials. Nutrients.

[4] de Borst M.H., Navis G. (2016). Sodium intake, RAAS-blockade and progressive renal disease. Pharmacol Res.

[5] MacKinnon H.J., Wilkinson T.J., Clarke A.L. (2018). The association of physical function and physical activity with all-cause mortality and adverse clinical outcomes in nondialysis chronic kidney disease: a systematic review. Ther Adv Chronic Dis.

[6] Cedillo-Couvert E.A., Ricardo A.C., Chen J.S. (2018). Self-reported medication adherence and CKD progression. Kidney Int Rep.

[7] Butler J.A., Roderick P., Mullee M., Mason J.C., Peveler R.C. (2004). Frequency and impact of nonadherence to immunosuppressants after renal transplantation: a systematic review. Transplantation.

[8] Ricardo A.C., Anderson C.A., Yang W. (2015). Healthy lifestyle and risk of kidney disease progression, atherosclerotic events, and death in CKD: findings from the Chronic Renal Insufficiency Cohort (CRIC) Study. Am J Kidney Dis.

[9] Bundy J.D., Bazzano L.A., Xie D.W. (2018). Self-reported tobacco, alcohol, and illicit drug use and progression of chronic kidney disease. Clin J Am Soc Nephrol.

[10] Ikizler T.A., Burrowes J.D., Byham-Gray L.D. (2020). KDOQI clinical practice guideline for nutrition in CKD: 2020 Update. Am J Kidney Dis.

[11] Choi N.G., Sullivan J.E., DiNitto D.M., Kunik M.E. (2019). Associations between psychological distress and health-related behaviors among adults with chronic kidney disease. Prev Med.

[12] Seng J.J.B., Tan J.Y., Yeam C.T., Htay H., Foo W.Y.M. (2020). Factors affecting medication adherence among pre-dialysis chronic kidney disease patients: a systematic review and meta-analysis of literature. Int Urol Nephrol.

[13] Palmer S.C., Vecchio M., Craig J.C. (2013). Prevalence of depression in chronic kidney disease: systematic review and meta-analysis of observational studies. Kidney Int.

[14] Loosman W.L., Rottier M.A., Honig A., Siegert C.E.H. (2015). Association of depressive and anxiety symptoms with adverse events in Dutch chronic kidney disease patients: a prospective cohort study. BMC Nephrol.

[15] de Ridder D., Geenen R., Kuijer R., van Middendorp H. (2008). Psychological adjustment to chronic disease. Lancet.

[16] Hudson J.L., Moss-Morris R. (2019). Treating illness distress in chronic illness: integrating mental health approaches with illness self-management. Eur Psychol.

[17] Kroenke K., Wu J.W., Yu Z.S. (2016). Patient Health Questionnaire Anxiety and Depression Scale: initial validation in three clinical trials. Psychosom Med.

[18] Paine N.J., Bacon S.L., Bourbeau J. (2019). Psychological distress is related to poor health behaviours in COPD and non-COPD patients: evidence from the CanCOLD study. Respir Med.

[19] Tonelli M., Wiebe N., Guthrie B. (2015). Comorbidity as a driver of adverse outcomes in people with chronic kidney disease. Kidney Int.

[20] Detweiler-Bedell J.B., Friedman M.A., Leventhal H., Miller I.W., Leventhal E.A. (2008). Integrating co-morbid depression and chronic physical disease management: identifying and resolving failures in self-regulation. Clin Psychol Rev.

[21] Shin J.K., Chiu Y.L., Choi S., Cho S., Bang H. (2012). Serious psychological distress, health risk behaviors, and diabetes care among adults with type 2 diabetes: the California Health Interview Survey 2007. Diabetes Res Clin Pract.

[22] Sumlin L.L., Garcia T.J., Brown S.A. (2014). Depression and adherence to lifestyle changes in type 2 diabetes: a systematic review. Diabetes Educ.

[23] Gebrie M.H., Ford J. (2019). Depressive symptoms and dietary non-adherence among end stage renal disease patients undergoing hemodialysis therapy: systematic review. BMC Nephrol.

[24] Kurita N., Wakita T., Ishibashi Y. (2020). Association between health-related hope and adherence to prescribed treatment in CKD patients: multicenter cross-sectional study. BMC Nephrol.

[25] von Elm E., Altman D.G., Egger M. (2007). The Strengthening the Reporting of Observational Studies in Epidemiology (STROBE) statement: guidelines for reporting observational studies. Lancet.

[26] Cardol C.K., Van Middendorp H., Dusseldorp E. (2023). eHealth to improve psychological functioning and self-management of people with chronic kidney disease: a randomized controlled trial. Psychosom Med.

[27] Hays R.D., Sherbourne C.D., Mazel R.M. (1993). The RAND 36-item health survey 1.0. Health Econ.

[28] Kroenke K., Spitzer R.L., Williams J.B.W. (2001). The PHQ-9: validity of a brief depression severity measure. J Gen Intern Med.

[29] Spitzer R.L., Kroenke K., Williams J.B.W., Löwe B. (2006). A brief measure for assessing generalized anxiety disorder: the GAD-7. Arch Intern Med.

[30] Chilcot J., Hudson J.L., Moss-Morris R. (2018). Screening for psychological distress using the Patient Health Questionnaire Anxiety and Depression Scale (PHQ-ADS): initial validation of structural validity in dialysis patients. Gen Hosp Psychiatry.

[31] Linting M., van der Kooij A. (2012). Nonlinear principal components analysis with CATPCA: a tutorial. J Pers Assess.

[32] Wendel-Vos G.C., Schuit A.J., Saris W.H., Kromhout D. (2003). Reproducibility and relative validity of the short questionnaire to assess health-enhancing physical activity. J Clin Epidemiol.

[33] Knobel H., Alonso J., Casado J.L. (2002). Validation of a simplified medication adherence questionnaire in a large cohort of HIV-infected patients: the GEEMA Study. AIDS.

[34] WHO Consultation on Obesity, World Health Organization Obesity: preventing and managing the global epidemic: report of a WHO consultation. World Health Organization. https://apps.who.int/iris/handle/10665/42330.

[35] Hoang D., Kristoffersen I., Li I.W. (2019). All in the mind? Estimating the effect of mental health on health behaviours. Soc Sci Med.

[36] Montez-Rath M.E., Winkelmayer W.C., Desai M. (2014). Addressing missing data in clinical studies of kidney diseases. Clin J Am Soc Nephrol.

[37] Konel J.M., Warsame F., Ying H. (2018). Depressive symptoms, frailty, and adverse outcomes among kidney transplant recipients. Clin Transplant.

[38] Belaiche S., Décaudin B., Dharancy S., Noel C., Odou P., Hazzan M. (2017). Factors relevant to medication non-adherence in kidney transplant: a systematic review. Int J Clin Pharm.

[39] Maes S., Leventhal H., de Ridder D., Endler M.Z.N.S. (1996). Handbook of Coping: Theory, Research, Applications.

[40] Cardol C.K., Boslooper-Meulenbelt K., Van Middendorp H., Meuleman Y., Evers A.W.M., van Dijk S. (2022). Psychosocial barriers and facilitators for adherence to a healthy lifestyle among patients with chronic kidney disease: a focus group study. BMC Nephrol.

[41] Schrauben S.J., Hsu J.Y., Wright Nunes J. (2019). Health behaviors in younger and older adults with CKD: results from the CRIC study. Kidney Int Rep.

[42] Sawchuk C.N., Olatunji B.O. (2011). Anxiety, health risk factors, and chronic disease. Am J Lifestyle Med.

[43] VanderWeele T.J. (2019). Principles of confounder selection. Eur J Epidemiol.

[44] Galobardes B., Lynch J., Smith G.D. (2007). Measuring socioeconomic position in health research. Br Med Bull.

[45] Lieb M., Hepp T., Schiffer M., Opgenoorth M., Erim Y. (2020). Accuracy and concordance of measurement methods to assess non-adherence after renal transplantation—a prospective study. BMC Nephrol.

[46] Warren J.M., Ekelund U., Besson H. (2010). Assessment of physical activity—a review of methodologies with reference to epidemiological research: A report of the exercise physiology section of the European Association of Cardiovascular Prevention and Rehabilitation. Eur J Cardiovasc Prev Rehabil.

[47] González A.M., Gutman T., Lopez-Vargas P. (2020). Patient and caregiver priorities for outcomes in CKD: A multinational nominal group technique study. Am J Kidney Dis.

[48] van der Willik E.M., Hemmelder M.H., Bart H.A.J. (2021). Routinely measuring symptom burden and health-related quality of life in dialysis patients: first results from the Dutch registry of patient-reported outcome measures. Clin Kidney J.

[49] Bos-Touwen I., Schuurmans M., Monninkhof E.M. (2015). Patient and disease characteristics associated with activation for self-management in patients with diabetes, chronic obstructive pulmonary disease, chronic heart failure and chronic renal disease: a cross-sectional survey study. PLoS One.

[50] Chung Y.C., Yeh M.L., Liu Y.M. (2017). Effects of intradialytic exercise on the physical function, depression and quality of life for haemodialysis patients: a systematic review and meta-analysis of randomised controlled trials. J Clin Nurs.

[51] Firth J., Marx W., Dash S. (2019). The effects of dietary improvement on symptoms of depression and anxiety: a meta-analysis of randomized controlled trials. Psychosom Med.

[52] Cukor D., Ver Halen N.V., Asher D.R. (2014). Psychosocial intervention improves depression, quality of life, and fluid adherence in hemodialysis. J Am Soc Nephrol.

[53] Lazarus R.S., Folkman S. (1984).

