# Supplementary material for: Psychological Distress and Self-Management in CKD: A Cross-Sectional Study
Source: Kidney Med. 2023 Aug 11;5(10):100712. doi: 10.1016/j.xkme.2023.100712 (PMC10518713; doi:10.1016/j.xkme.2023.100712)
Supplement: Supplementary File (PDF) — Item S1; Tables S1-S5. [file mmc1.docx]

**Item S1.** E-GOAL Study Group

Drs. Cinderella K. Cardol, Leiden University

Dr. Henriët van Middendorp, Leiden University

Dr. Paul J. M. van der Boog, Leiden University Medical Center

Prof. Dr. Gerjan Navis, University Medical Center Groningen

Prof. Dr. Luuk B. Hilbrands, Radboud university medical center

Dr. Yvo W. J. Sijpkens, Haaglanden Medical Center

Dr. Yvette Meuleman, Leiden University Medical Center

Drs. Karin Boslooper-Meulenbelt, University Medical Center Groningen

Dr. Sasja Huisman, Leiden University Medical Center

Jan A. M. Luijten, patient research partner

Carla van Dorp, patient research partner

Prof. dr. Friedo W. Dekker, Leiden University Medical Center

Dr. Joris I. Rotmans, Leiden University Medical Center

Prof. Dr. Ton J. Rabelink, Leiden University Medical Center

Prof. Dr. Niels H. Chavannes, Leiden University Medical Center

Dr. Jacob K. Sont, Leiden University Medical Center

Dr. Eduard M. Scholten, Haaglanden Medical Center

Prof. Dr. Andrea W. M. Evers, Leiden University

Dr. Sandra van Dijk, Leiden University

**Table S1.** *Patient characteristics by subgroups of adherence and non-adherence to self-management recommendations*

| Characteristic | Completely adherent (*n*=137) | ≥1*Non-adherent (*n*=321)^a^ |
| --- | --- | --- |
| **Socio-demographic characteristics** |  |  |
| Age, y | 59.4±11.9 | 58.2±12.6 |
| Male sex, *n* (%) | 84 (61.3) | 202 (62.9) |
| Born in the Netherlands, *n* (%) | 132 (96.4) | 300 (93.5) |
| Married/partnered, *n* (%) | 116 (84.7) | 248 (77.3) |
| Having children, *n* (%) | 94 (68.6) | 235 (73.2) |
| Lower education^b,c^, *n* (%) | 57 (41.6) | 169 (52.6) |
| Unemployed, *n* (%) | 69 (50.4) | 162 (50.5) |
| **Disease and treatment characteristics** |  |  |
| Kidney transplant recipient, *n* (%) | 99 (72.3) | 216 (67.3) |
| Time since last kidney transplantation^d,e^, y | 10.0±7.9 | 9.3±8.3 |
| History of dialysis, *n* (%) | 50 (36.5) | 125 (38.0) |
| Multimorbidity^e^ | 59 (43.1) | 163 (50.8) |
| Diabetes mellitus^e^, *n* (%) | 17 (12.4) | 53 (16.5) |
| Cardiovascular disease^e^, *n* (%) | 21 (15.3) | 49 (15.3) |
| Hypertension^a^, *n* (%) | 33 (24.1) | 111 (34.6) |
| eGFR, mL/min/1.73 m2 | 52.5±16.5 | 49.3±18.0 |
| Office SBP, mm Hg^f^ | 131.3±15.8 | 134.6±15.7 |
| Office DBP, mm Hg^f^ | 76.0±9.6 | 78.7±9.6 |
| Treatment history for psychological complaints^e^, *n* (%) | 30 (21.9) | 96 (29.9) |
| Current treatment for psychological complaints^e^, *n* (%) | 2 (1.5) | 20 (6.2) |
| Physical HRQoL^a^ | 46.0±9.7 | 42.0±11.0 |
| Mental HRQoL^c^ | 51.1±9.8 | 37.1±11.1 |

*Notes.* Continuous variables are presented as mean±SD for normally distributed variables and as median[IQR] for skewed variables; DBP = diastolic blood pressure; eGFR=estimated glomerular filtration rate; HRQoL=health-related quality of life; SBP=systolic blood pressure. ^a^2 unknown; ^b^Lower education includes primary, pre-vocational, and vocational education; Higher education includes advanced secondary and tertiary education; ^c^3 unknown; ^d^Only for kidney transplant recipients, *n*=316; ^e^1 unknown; ^f^26 unknown.

Below, the results of the analyses can be found, repeated on the original dataset without multiple imputation (Tables S2-S5).

**Table S2.** *Linear regression of psychological distress and CKD self-management*

|  | Crude | | Adjusted^a^ | |
| --- | --- | --- | --- | --- |
|  | Coeff.^b^ (95% CI) | *p* | Coeff.^b^ (95% CI) | *p* |
| Dietary adherence | -0.19 (-0.29 to -0.10) | <0.001 | -0.14 (-0.24 to -0.05) | 0.003 |
| Physical activity | -0.10 (-0.19 to -0.01) | 0.032 | -0.12 (-0.21 to -0.02) | 0.022 |
| Medication adherence | -0.19 (-0.28 to -0.10) | <0.001 | -0.14 (-0.24 to -0.05) | 0.004 |
| Body mass index | 0.11 (0.02 to 0.21) | 0.014 | 0.09 (-0.01 to 0.19) | 0.066 |
| Smoking | 1.03 (0.99 to 1.07) | 0.194 | 1.04 (0.99 to 1.08) | 0.134 |

*Notes.* CI=Confidence Interval; Coeff.=Regression Coefficient. ^a^Adjusted for age, sex, education level, marital status, comorbidities, and kidney function (estimated glomerular filtration rate); ^b^Beta for continuous dependent variables, odds ratio for dichotomous variable.

**Table S3.** *Linear regression of depressive symptoms and CKD self-management*

|  | Crude | | Adjusted^a^ | |
| --- | --- | --- | --- | --- |
|  | Coeff.^b^ (95% CI) | *p* | Coeff.^b^ (95% CI) | *p* |
| Dietary adherence | -0.20 (-0.29 to -0.11) | <0.001 | -0.15 (-0.24 to -0.05) | 0.003 |
| Physical activity | -0.13 (-0.22 to -0.04) | 0.007 | -0.14 (-0.24 to -0.04) | 0.005 |
| Medication adherence | -0.18 (-0.27 to -0.09) | <0.001 | -0.14 (-0.24 to -0.05) | 0.003 |
| Body mass index | 0.12 (0.03 to 0.21) | 0.012 | 0.09 (-0.01 to 0.18) | 0.082 |
| Smoking | 1.06 (0.99 to 1.14) | 0.088 | 1.07 (1.00 to 1.16) | 0.066 |

*Notes.* CI=Confidence Interval; Coeff.=Regression Coefficient. ^a^Adjusted for age, sex, education level, marital status, comorbidities, and kidney function (estimated glomerular filtration rate); ^b^Beta for continuous dependent variables, odds ratio for dichotomous variable.

**Table S4.** *Linear regression of anxiety symptoms and CKD self-management*

|  | Crude | | Adjusted^a^ | |
| --- | --- | --- | --- | --- |
|  | Coeff.^b^ (95% CI) | *p* | Coeff.^b^ (95% CI) | *p* |
| Dietary adherence | -0.17 (-0.26 to -0.08) | <0.001 | -0.12 (-0.21 to -0.02) | 0.014 |
| Physical activity | -0.05 (-0.14 to 0.05) | 0.327 | -0.06 (-0.16 to 0.04) | 0.241 |
| Medication adherence | -0.17 (-0.26 to -0.08) | <0.001 | -0.12 (-0.21 to -0.02) | 0.015 |
| Body mass index | 0.06 (-0.03 to 0.15) | 0.207 | 0.05 (-0.05 to 0.14) | 0.328 |
| Smoking | 1.03 (0.94 to 1.12) | 0.522 | 1.04 (0.95 to 1.14) | 0.387 |

*Notes.* CI=Confidence Interval; Coeff.=Regression Coefficient. ^a^Adjusted for age, sex, education level, marital status, comorbidities, and kidney function (estimated glomerular filtration rate); ^b^Beta for continuous dependent variables, odds ratio for dichotomous variable.

**Table S5.** *Ordinal logistic regression of CKD self-management index (CSI) by psychological distress, depressive, and anxiety symptoms*

| CSI | Crude | | Adjusted^a^ | |
| --- | --- | --- | --- | --- |
|  | OR^b^ (95% CI) | *p* | OR^b^ (95% CI) | *p* |
| Psychological distress | 1.05 (1.03 to 1.08) | <0.001 | 1.04 (1.02 to 1.07) | 0.001 |
| Depressive symptoms | 1.11 (1.06 to 1.16) | <0.001 | 1.09 (1.04 to 1.14) | <0.001 |
| Anxiety symptoms | 1.08 (1.03 to 1.13) | 0.001 | 1.06 (1.01 to 1.11) | 0.018 |

*Notes.* OR=Odds Ratio; CI=Confidence Interval. ^a^Adjusted for age, sex, education level, marital status, comorbidities, and kidney function (estimated glomerular filtration rate).
